# Staged patellar tendon reconstruction using doubled bone-patellar tendon-bone allograft for infected patellar tendon rupture: a rare case report of three years follow-up

**DOI:** 10.1186/s40634-021-00334-1

**Published:** 2021-02-18

**Authors:** Hyung Suk Choi, Byung-Woong Jang, Dong-Il Chun, Yong Beom Kim, Gi-Won Seo, Jinyeong Hwang, Byung Ill Lee

**Affiliations:** 1 Department of Orthopaedic Surgery, Soonchunhyang University Hospital Seoul, 59, Daesagwan-ro, Yongsan-gu, Seoul, 04401 Korea; 2 Department of Orthopaedic Surgery, Soonchunhyang University Hospital Gumi, Gumi, Korea; 3 Department of Orthopedic Surgery, Smarton Hospital, Bucheon, Korea

**Keywords:** Tendon injury, Patellar tendon, Rupture

## Abstract

**Background:**

Patellar tendon rupture is a relatively rare injury that usually requires surgical treatment. The optimal therapeutic strategy is still controversial, especially when either concomitant patellar tendon infection or soft tissue infection surrounds the patellar tendon. Until recently, most reported reconstruction methods are extensive and difficult to apply because of the poor condition of the soft tissue surrounding the patellar tendon.

**Case presentation:**

A 19-year-old male patient presented to our clinic three weeks following a motorcycle accident. There was a 5 x 4 cm sized skin defect with soft tissue infection below the inferior pole of patella. We performed a staged patellar tendon reconstruction using a doubled bone-patellar tendon-bone allograft (BPTB) to the infected patellar tendon rupture, following local random fasciocutaneous flap and split-thickness skin graft. Three months following surgery, the patient was able to perform an active knee motion with no extension lag and excellent clinical functional result.

**Discussion and conclusions:**

Our technique introduced in this specific case is a relatively simple method to reconstruct chronic patellar tendon defects with limited incision exposing only the patellar tendon areas. We expect it can be less invasively performed on patients who have a soft tissue problem and cannot have extensive surgery.

## Introduction

Patellar tendon rupture is a relatively rare injury and often overlooked if a careful examination is not carried out during the acute phase. Traumatic patellar tendon rupture represents 0.6% of the musculoskeletal tendinous injuries in the general population [8]. In the United States, patellar tendon ruptures tend to affect less than 0.5% of the population per year with peak age incidence at around 40 years [24]. Males are more commonly affected than are females because males are more physically active and more susceptible to ligamentous rupture. Biomechanically, it is known that the patellar tendon undergoes a stress that is 3.2 times the body weight when going up the stairs. In the absence of other conditions, rupture of the patellar tendon can occur at about 17.5 times the body weight [27]. Patellar tendon rupture may occur following several surgical procedures, such as total knee arthroplasty (TKA) or anterior cruciate ligament (ACL) reconstruction using autogenous bone-patellar tendon-bone (BPTB). It can also occur in patients with systemic diseases such as rheumatoid arthritis (RA), systemic lupus erythematosus (SLE), or chronic kidney disease (CKD) who can experience weakening of the collagen fiber.

There are many ways to treat patellar tendon ruptures. In the case of acute lesions, primary repair with or without augmentation is possible. On the other hand, if there is a defect that cannot be repaired primarily, there are options to reconstruct the patellar tendon using various types of grafting, including autograft or allograft [5, 7, 9, 13, 16, 18, 23]. The surgical method of treatment should be selected based on the rupture pattern and duration of the injury, as well as the factors of the patient's condition that may affect clinical outcomes and functions [1, 3, 10].

Delayed patellar tendon reconstruction presents challenges caused by retraction of the patella proximally and scar formation of the surrounding soft tissues [22]. Several surgical techniques have been reported describing delayed reconstruction of chronic patellar tendon rupture. However, which of various approaches would be the ideal surgical method is still controversial [6, 11]. Most reported reconstruction methods are extensive and difficult to apply because of the poor condition of the soft tissue surrounding the patellar tendon. In addition, deep infection following patella tendon injury can be a disastrous problem, which may be difficult to treat successfully.

In this case report, we describe successful mid-term follow-up results of a case of staged patellar tendon reconstruction, following regional flap surgery that had been performed for soft tissue defect on the anterior knee, using doubled BPTB allograft in a patient who had sustained infected chronic patellar tendon rupture. To the best of our knowledge, this is the first report of a staged patellar tendon reconstruction using doubled BPTB allograft.

## Case presentation

**Fig. 1.**
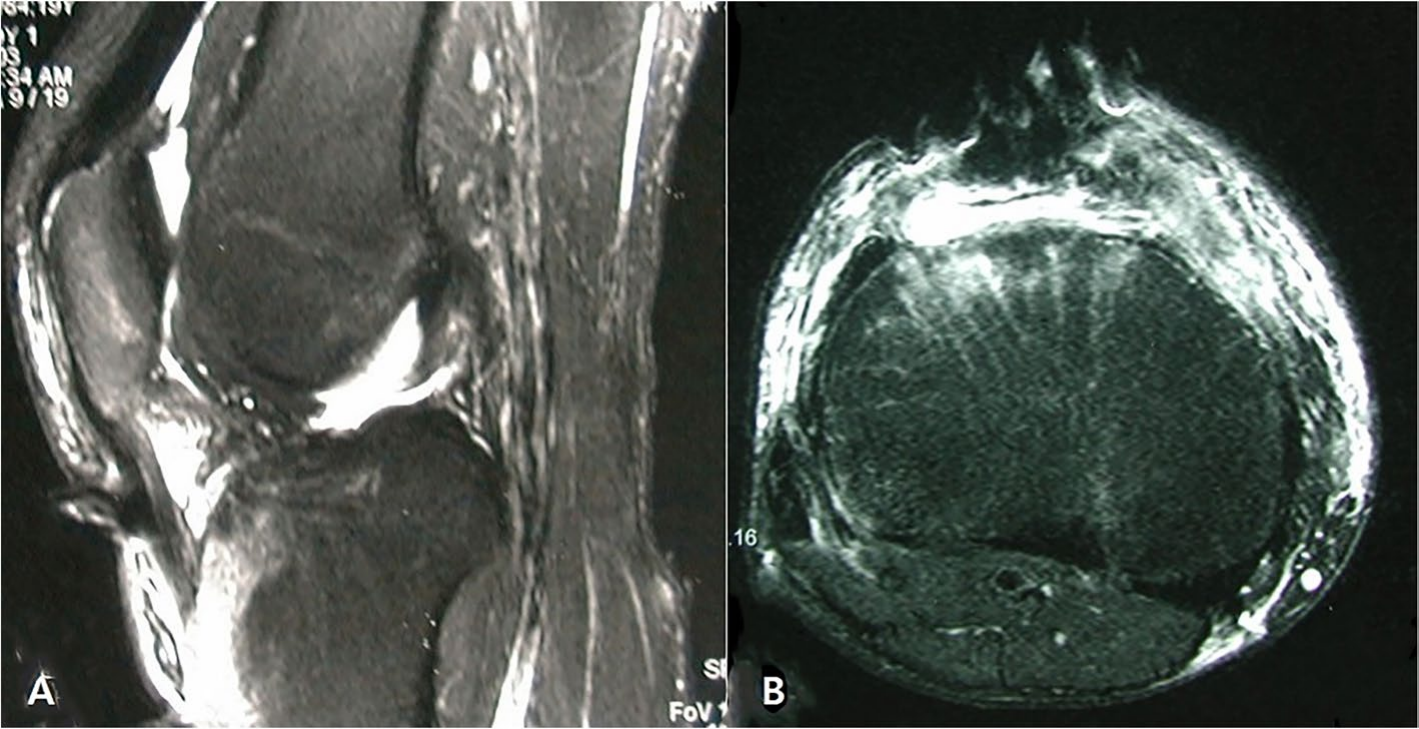
Initial T2-weighted MRI images showing patellar tendon rupture observed as defect on the distal part. **a** Sagittal image. **b** Axial image

Patient has provided informed consent for publication of the case. A 19-year-old male patient presented to our clinic three weeks following a motorcycle accident. The height and weight of the patient were 176 cm and 72 kg (body mass index 23.2 kg/m^2^), and he had no medical history. The patient had undergone initial treatment at another hospital, consisting of two irrigation and debridement procedures under general anesthesia. Wound culture revealed pseudomonas aeruginosa, and they had administered ciprofloxacin for 2 weeks. On the initial MRI, the partially ruptured patellar tendon was identified (Fig. 1a and b), but uncontrolled persistent infection resulted in defects in the patellar tendon by the time he had come to visit our clinic.

On examination, there was a 5 x 4 cm sized skin defect below the inferior pole of patella, and the patellar tendon was exposed. We observed a brown-colored purulent discharge, redness, and local heating. The patient was unable to actively extend the knee against gravity, which suggested grade II motor power.

**Fig. 2.**
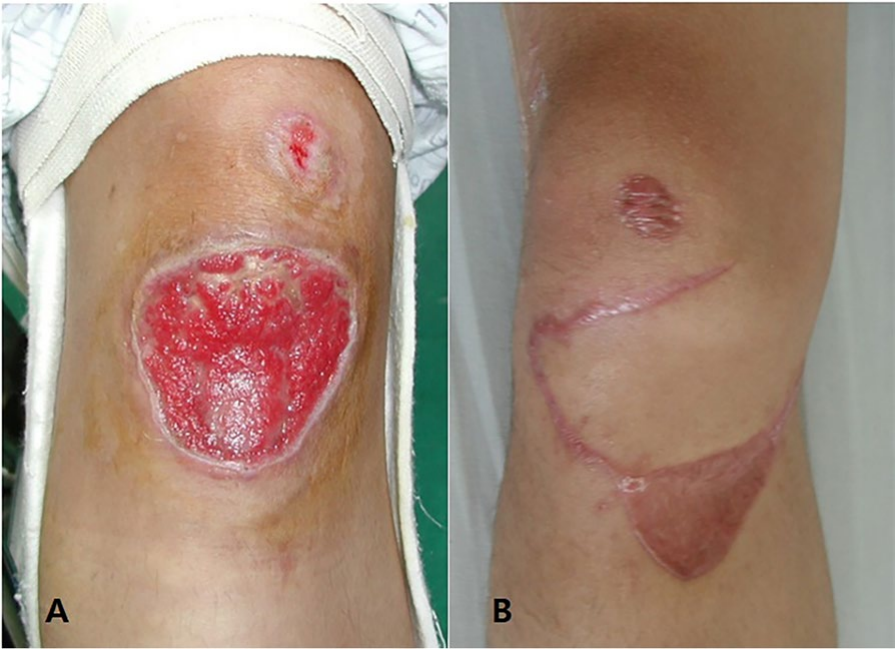
**a** Clinical image showing a clear wound bed with fresh granulation tissue in the infrapatellar skin defect area. **b** Clinical image with controlled infection showing complete wound healing after local random fasciocutaneous flaps and split-thickness skin grafts

We decided on staged management to include eradication of the infection, with soft tissue coverage, to be followed by patellar tendon reconstruction. We obtained a clean soft tissue wound status following four weeks of repeated debridement with intravenous antibiotic administration (ciprofloxacin) and the formation of a good granulation tissue wound bed for flap coverage (Fig. 2a). In the laboratory findings, ESR was slightly elevated to 27 mm/hr (normal range, 0 ~ 15 mm/hr). Other infection markers were within normal range, with a WBC of 7.8 x 10^3^/mL (normal range, 4.0 ~ 10.0 x 10^3^/ml), and a CRP of 0.3 mg/dL (normal range, 0 ~ 0.5 mg/dL). We used a local random fasciocutaneous flap and split-thickness skin graft to close the open wounds first. One month following complete wound healing of the flap surgery (Fig. 2b), we performed a staged patellar tendon reconstruction using doubled BPTB allograft.

## Surgical technique

**Fig. 3.**
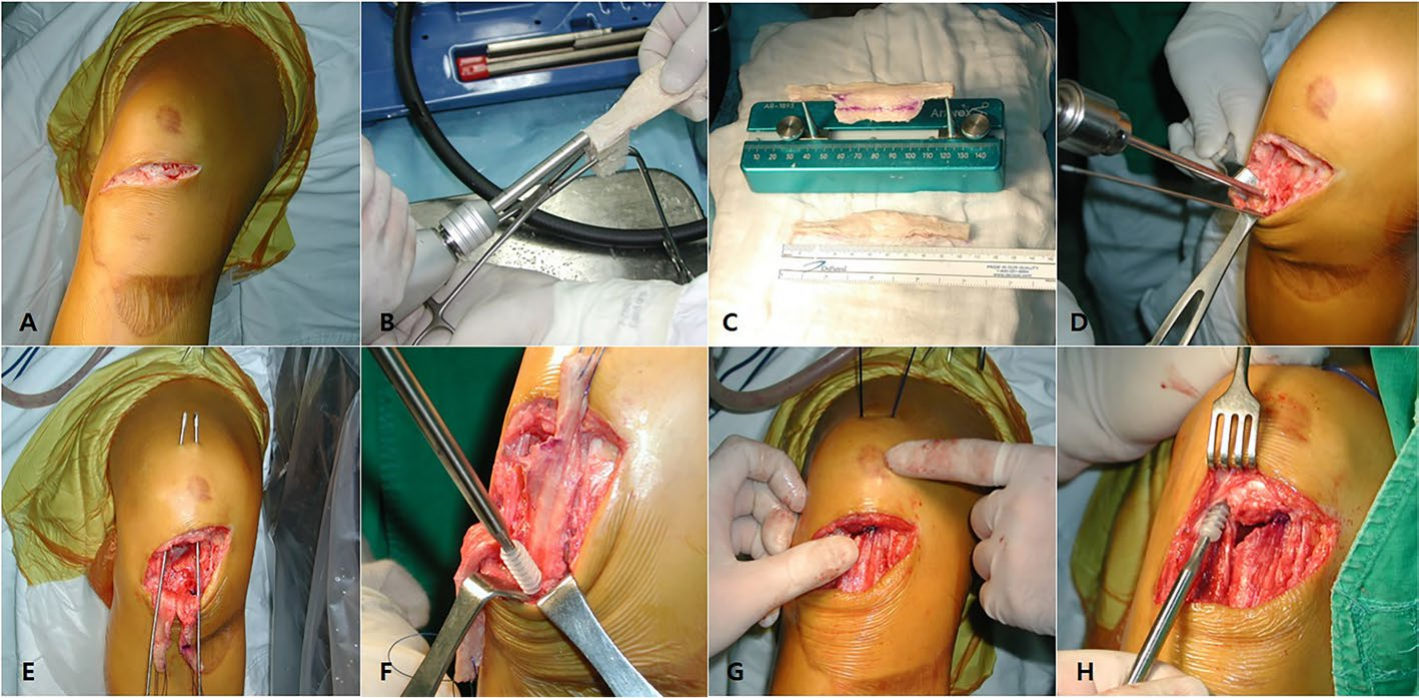
**a-h** Operative procedure of patellar tendon reconstruction with double BTB allograft

**Fig. 4.**
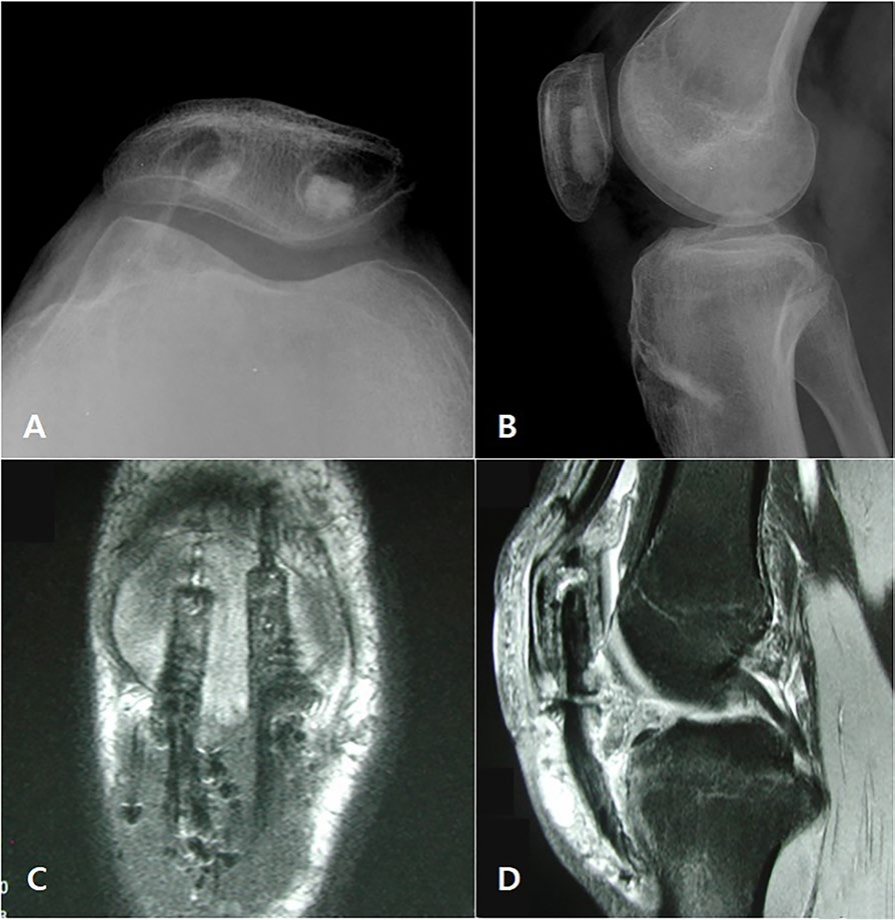
Postoperative images. **a** Skyline view of plain radiograph (**b**) Lateral view of plain radiograph. **c** T2 weighted sagittal MRI. **d** T1 weighted coronal MRI

We performed the surgery under general anesthesia. We incised the oblique skin about 6 cm along the previous skin flap scar and the exposed patellar tendon. The remnant tissues of patellar tendon in the tibial tuberosity had been completely absorbed and showed no traces. At the inferior pole of the patella where the patellar tendon inserts, fibrotic tissue remained hypertrophic, which left about a 1-cm of remnant that had been adherent to the surrounding tissue. We removed all soft tissue where the patella tendon was attached and prepared the bone bed. Then we designed two BPTB allografts that were 8.4 mm x 25 mm in size. We shaped them into round shaped bone plugs, using a round shaped saw. We inserted two guide pins into the medial and the lateral side of the tibia at about a 45-degree angle longitudinal to the axis of tibia. Then, we made a tibial bone tunnel, using a 9 mm diameter reamer. We drove one side of the graft bone blocks through the bone tunnel and affixed it with a 9 x 25 mm absorbable interference screw (Arthrex, Naples, FL). In the inferior pole of the patella, we inserted the guide pins into the medial and the lateral convergence, and we made a patellar bone tunnel about 39 mm in depth with a 9-mm diameter reamer. After passing the bone block through the tunnel, we performed a rigid fixation, using a 9 x 30 mm absorbable interference screw (Arthrex, Naples, FL). We measured the length of the opposite patellar tendon (40mm) on a lateral x-ray of knee flexed by 60 degrees before surgery to accurately match the patellar height. During surgery, we tried to insert a graft of the same length as the measured value while the knee was flexed by 60 degrees. (Fig. 3a-h) A postoperative X-ray confirmed a reasonable patellar height, and an MRI confirmed graft continuity and tension (Fig. 4a-d).

## Rehabilitation

**Fig. 5.**
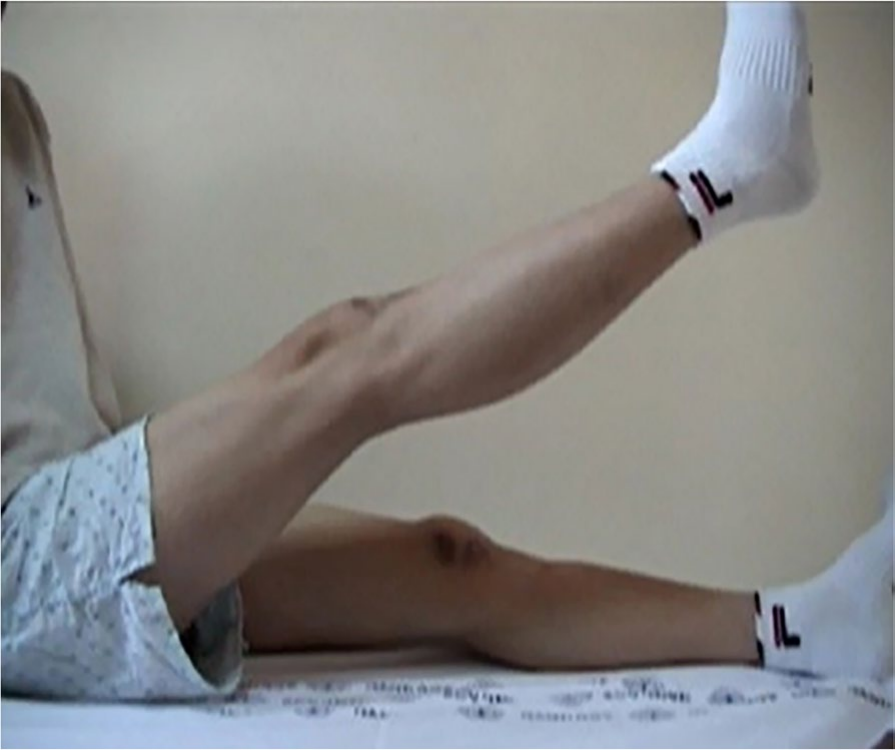
A photograph of the patient with active straight leg raise, three months following surgery

After flap surgery, cylinder splint was applied for two weeks to stabilize the wound. Patient started passive range of motion exercise using continuous passive motion (CPM) device one week after surgery. After the knee joint range of motion was sufficiently recovered, a stepwise patellar tendon reconstruction was performed. After patellar tendon reconstruction, immediate isometric quadriceps exercise was permitted as tolerated, and we applied a drop-out cast to be worn for 3 weeks to maintain fixation. Three weeks after surgery, the patient was made to perform 45 degrees of passive and active-assisted knee joint range-of-motion exercise. This was advanced in 10- to 15-degrees increments each week. We started active range-of-motion 6 weeks following surgery. Three months following surgery, the patient was able to perform an active knee extension with no extension lag at a 130-degree flexion angle (Fig. 5). He was able to return to sports activity after six months with no specific limitation. Three years after surgery, the patient showed excellent clinical results with an IKDC score of 92 and Lysholm score of 95. The length of the patellar tendon was well maintained on the last follow-up x-ray, which also showed the bone plug to be well incorporated.

## Discussion and findings

Extensor mechanism injuries include patellar tendon rupture, patellar dislocation, patellar fracture, tibial tubercle avulsion fracture, and quadriceps tendon rupture. Among these, patellar tendon rupture is an uncommon injury. It accounts for the 3rd frequent cause of adult native extensor mechanism ruptures after patella fractures and quadriceps tendon ruptures [2]. The causes of patellar tendon rupture include 1) Indirect trauma, 2) Preexisting systemic disorders (RA, SLE, CKD, diabetes mellitus), 3) long-term use of systemic corticosteroid, local steroid injection, 4) Surgical procedure-related complications (TKA, ACL reconstruction with BPTB autograft), and 5) direct trauma or local infection. But its real incidence and prevalence are not yet known [19, 25].

Patellar tendon rupture usually requires surgical treatment including repair, repair with augmentation, or reconstruction. Treatment goals of patellar tendon injury include the restoration of the extensor mechanism, both functionally and structurally, which allows for active knee joint extension and return to pre-injury level of activities. The management of patellar tendon ruptures remains a difficult therapeutic challenge, because complications such as changing patellar height, quadriceps contracture, and adhesions may occur. Furthermore, treatment becomes more difficult and controversial when infection of the patellar tendon or of the soft tissue that surrounds the patellar tendon occurs.

After a patellar tendon injury, it is best to repair it as soon as possible to restore the extensor mechanism of the knee. However, reconstruction is necessary in cases where primary repair is not possible, such as large defects, undetected injury, or failure of the initial treatment. However, established reconstruction methods require the wide exposure of the patella and the quadriceps muscles. Therefore, this is not suitable for patients with soft tissue problems.

Allograft is a surgical option that can be used when there is a large defect in the patellar tendon caused by extensor mechanism collapse after TKA, neglected patellar tendon injury, or sarcoma around the anterior knee [15, 21]. Allograft has the advantage of being less invasive because it can be performed with a relatively simple procedure, and there is no need to harvest tendons. However, when used extensively in bony reconstruction, there is the risk of fracture and a higher infection rate with autograft [4, 12]. In this case, we performed reconstruction using allograft after all wound defects and infections had resolved.

Among the various reconstruction allograft materials, the BPTB allograft has bone blocks on both sides, which provides initial fixation strength. ACL reconstruction using BPTB allograft has shown successful outcomes in young, high-demand, and active patients. Temponi et al. [25] reported a good surgical outcome by using contralateral BPTB autograft in chronic patellar tendon rupture. And reconstruction using BPTB allograft in patients with rheumatoid arthritis was introduced [20]. However, existing methods to affix the bone block cannot be free from the disadvantages of having patellar fracture and wide anterior knee involvement such as osteotomy of the patella [17, 26]. In this case we selected BPTB allograft and pulled out the BPTB allograft through the bone hole of patella to affix the graft to the patellar side. Since the patient in this case is a 19-year-old young and high-activity patient who has a sufficiently large size of the patella, doubled BPTB allograft was chosen for sufficient stable fixation power. This method obtains strong fixation with limited exposure in those patients in whom we cannot make an extensive incision because of previous flap surgery.

The position of the patella is one of the most important factors for normal knee joint function and stability. Among the anatomical alterations that affect the knee joint, patella alta is the one that is most associated with instability and recurrent dislocation [14]. Therefore, maintaining patellar height when reconstructing the patellar tendon is an important factor influencing the functional outcome. We measured the length of the patellar tendon on a 30-degree flexion lateral X-ray of the contralateral knee before surgery. With reference to the length of the opposite patellar tendon, we were able to reconstruct the tendon to an appropriate location without patellar alta or baja.

## Conclusion

In summary, the technique introduced in this case is a simple method to reconstruct chronic patellar tendon defects with limited incision exposing only the patellar tendon areas. The technique described above can be less invasively performed on patients who have a soft tissue problem and cannot have extensive surgery. We believe that the surgeon can achieve strong fixation and stability through this technique for patients with sufficient patella thickness and good bone stock. Consequently, it is a good way to restore knee function and strength. In the future, long-term studies using large data are needed to assess the outcomes and surgical techniques of these injuries.

## Abbreviations

BPTB: Bone-patellar tendon-bone
TKA: Total knee arthroplasty
ACL: Anterior cruciate ligament
RA: Rheumatoid arthritis
SLE: Systemic lupus erythematosus
CKD: Chronic kidney disease

## References

[1] Abdou YE (2014). Reconstruction of a chronic patellar tendon rupture with semitendinosus autograft. Arch Orthop Trauma Surg.

[2] Annunziata C, Ignacio E (2002). Patellar Tendon Rupture. E-Medicine.

[3] Ares O, Lozano LM, Medrano-Najera C, Popescu D, Martinez-Pastor JC, Segur JM (2014). New modified Achilles tendon allograft for treatment of chronic patellar tendon ruptures following total knee arthroplasty. Arch Orthop Trauma Surg.

[4] Bonnin M, Lustig S, Huten D (2016). Extensor tendon ruptures after total knee arthroplasty. Orthop Traumatol Surg Res.

[5] Cadambi A, Engh GA (1992). Use of a semitendinosus tendon autogenous graft for rupture of the patellar ligament after total knee arthroplasty. A report of seven cases. J Bone Joint Surg Am.

[6] Chen B, Li R, Zhang S (2012). Reconstruction and restoration of neglected ruptured patellar tendon using semitendinosus and gracilis tendons with preserved distal insertions: two case reports. Knee.

[7] Chiou HM, Chang MC, Lo WH (1997). One-stage reconstruction of skin defect and patellar tendon rupture after total knee arthroplasty. A new technique. J Arthroplasty.

[8] Clayton RA, Court-Brown CM (2008). The epidemiology of musculoskeletal tendinous and ligamentous injuries. Injury.

[9] Crossett LS, Sinha RK, Sechriest VF, Rubash HE (2002). Reconstruction of a ruptured patellar tendon with achilles tendon allograft following total knee arthroplasty. J Bone Joint Surg Am.

[10] Emerson RH, Head WC, Malinin TI (1994). Extensor mechanism reconstruction with an allograft after total knee arthroplasty. Clin Orthop Relat Res.

[11] Enad JG (1999). Patellar tendon ruptures. South Med J.

[12] Fujikawa K, Ohtani T, Matsumoto H, Seedhom BB (1994). Reconstruction of the extensor apparatus of the knee with the Leeds-Keio ligament. J Bone Joint Surg Br.

[13] Fukuta S, Kuge A, Nakamura M (2003). Use of the Leeds-Keio prosthetic ligament for repair of patellar tendon rupture after total knee arthroplasty. Knee.

[14] Greiwe RM, Saifi C, Ahmad CS, Gardner TR (2010). Anatomy and biomechanics of patellar instability. Oper Tech Sports Med.

[15] Hornicek FJ, Mnaymneh W, Lackman RD, Exner GU, Malinin TI (1998). Limb salvage with osteoarticular allografts after resection of proximal tibia bone tumors. Clin Orthop Relat Res.

[16] Lewis PB, Rue JP, Bach BR (2008). Chronic patellar tendon rupture: surgical reconstruction technique using 2 Achilles tendon allografts. J Knee Surg.

[17] Malhotra R, Garg B, Logani V, Bhan S (2008). Management of extensor mechanism deficit as a consequence of patellar tendon loss in total knee arthroplasty: a new surgical technique. J Arthroplasty.

[18] Milankov MZ, Miljkovic N, Stankovic M (2007). Reconstruction of chronic patellar tendon rupture with contralateral BTB autograft: a case report. Knee Surg Sports Traumatol Arthrosc.

[19] Nguene-Nyemb AG, Huten D, Ropars M (2011). Chronic patellar tendon rupture reconstruction with a semitendinosus autograft. Orthop Traumatol Surg Res.

[20] Palencia J, Alfayez SM, Alshammri AA, Serhan HS, Serro F, Alomar AZ (2016). Late reconstruction of the patellar tendon in rheumatoid arthritis using bone-patellar tendon-bone allograft. Int J Surg Case Rep.

[21] Rosenberg AG (2012). Management of extensor mechanism rupture after TKA. J Bone Joint Surg Br.

[22] Samagh SP, Huyke FA, Buchler L, Terry MA, Tjong VK (2018). Treatment of a neglected patellar tendon rupture with a modified surgical technique: ipsilateral semitendinosus autograft reconstruction with suture tape augmentation. Case Rep Orthop.

[23] Scuderi C (1958). Ruptures of the quadriceps tendon; study of twenty tendon ruptures. Am J Surg.

[24] Siwek CW, Rao JP (1981). Ruptures of the extensor mechanism of the knee joint. J Bone Joint Surg Am.

[25] Temponi EF, Camelo N, Tuteja S, Thaunat M, Daggett M, Fayard JM (2017). Reconstruction of chronic patellar tendon rupture with contralateral bone-tendon-bone autograft. Knee Surg Sports Traumatol Arthrosc.

[26] Zanotti RM, Freiberg AA, Matthews LS (1995). Use of patellar allograft to reconstruct a patellar tendon-deficient knee after total joint arthroplasty. J Arthroplasty.

[27] Zernicke RF, Garhammer J, Jobe FW (1977). Human patellar-tendon rupture. J Bone Joint Surg Am.

